# Supplementary material for: Phased nanopore assembly with Shasta and modular graph phasing with GFAse
Source: bioRxiv. 2023 Feb 22:2023.02.21.529152. Preprint. [Version 1] doi: 10.1101/2023.02.21.529152 (PMC9980101; doi:10.1101/2023.02.21.529152)
Supplement: Supplement 1 [file media-1.pdf]

Supplementary Figures and Tables

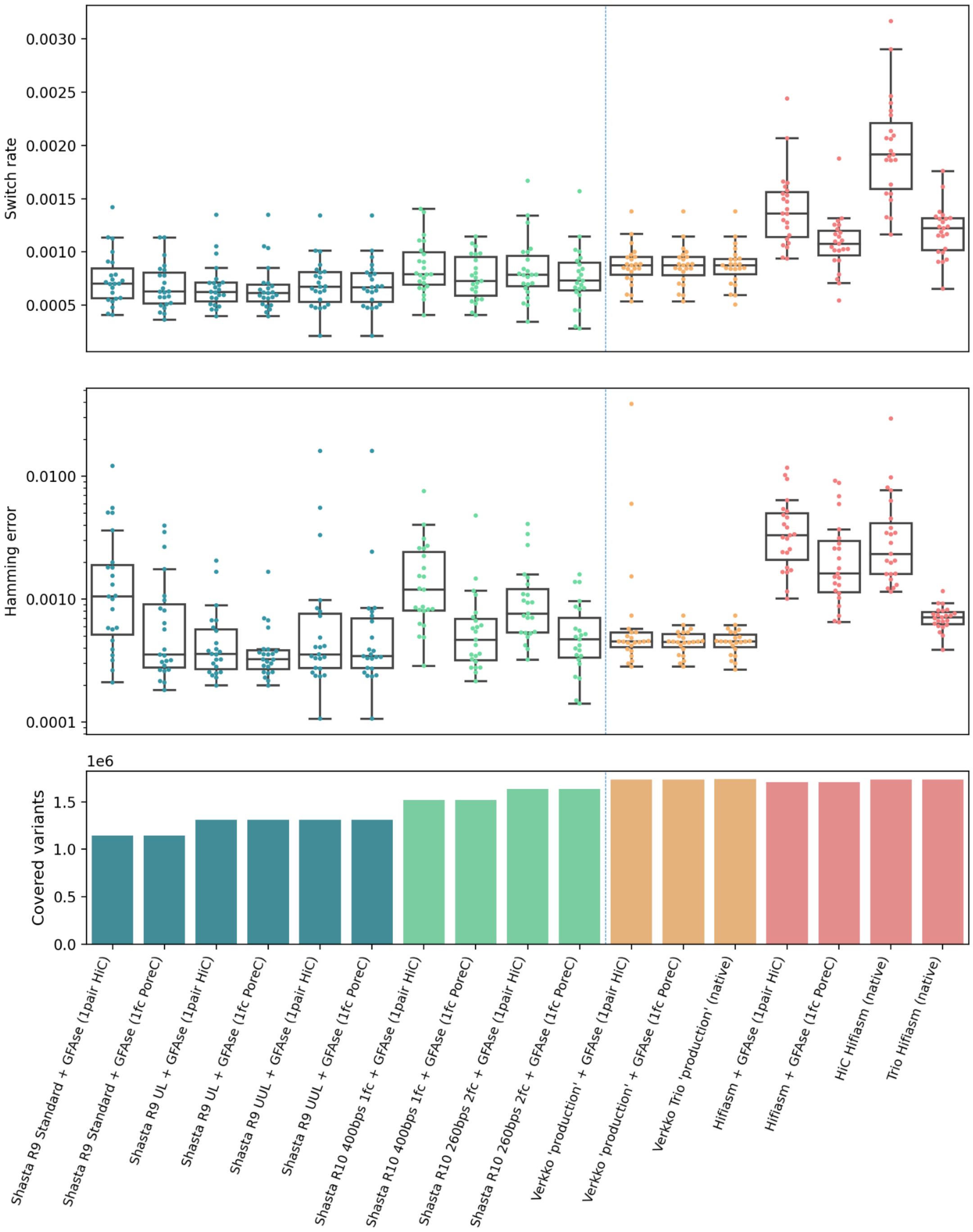

Supplementary Figure 1: Comparison of single pair HiC vs single flowcell PoreC (using GIAB StrandSeqANDTrio v4.2.1). Note that this truth set has fewer variants than that used in the main text.

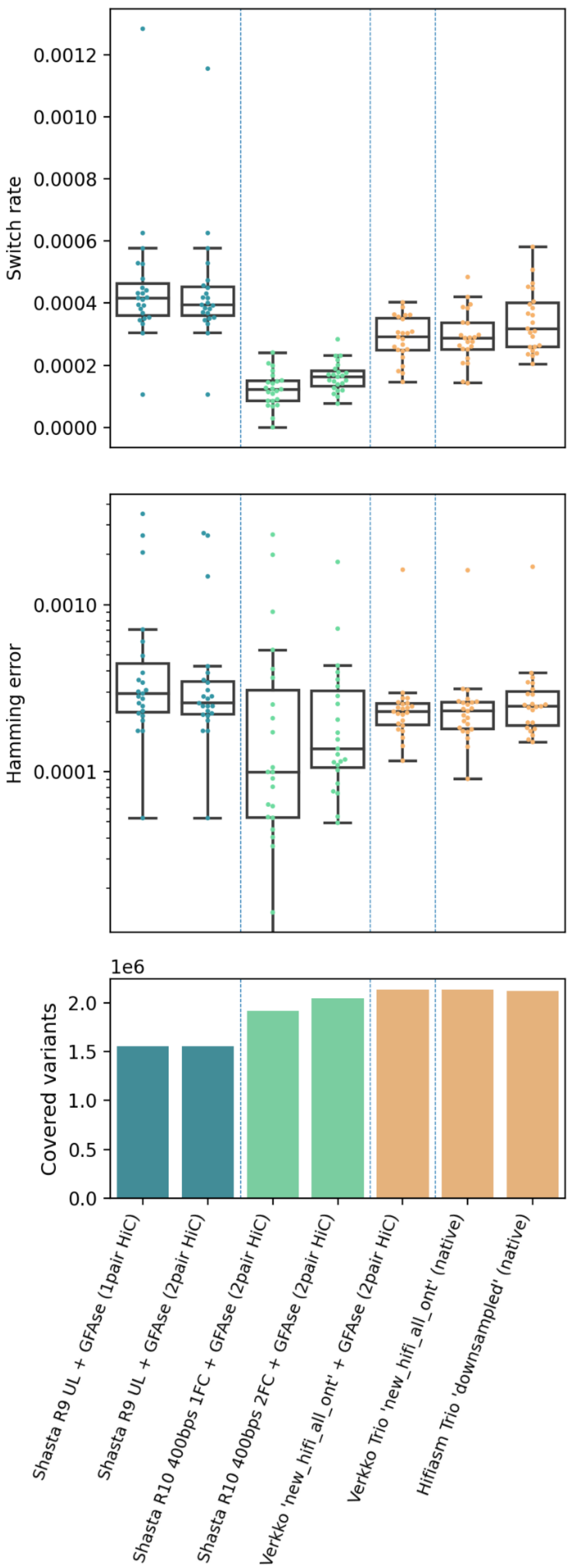

Supplementary Figure 2: HG005 evaluation using StrandSeq integrative phasing as a truth set.

		ONT		CCS		HiC
**Assembler**	**Label**	**Coverage**	**N50 (Kbp)**	**Coverage**	**N50 (Kbp)**	**Coverage**
**Shasta**	R9 UL	57.1	111			30
**Shasta**	R10 1FC	34.0	32			30
**Shasta**	R10 2FC	57.2	34			30
**Verkko Trio**	new_hifi_all_ont	185.8	81.2	33.7	16.06	30

Supplementary Table 1: HG005 assembly input data

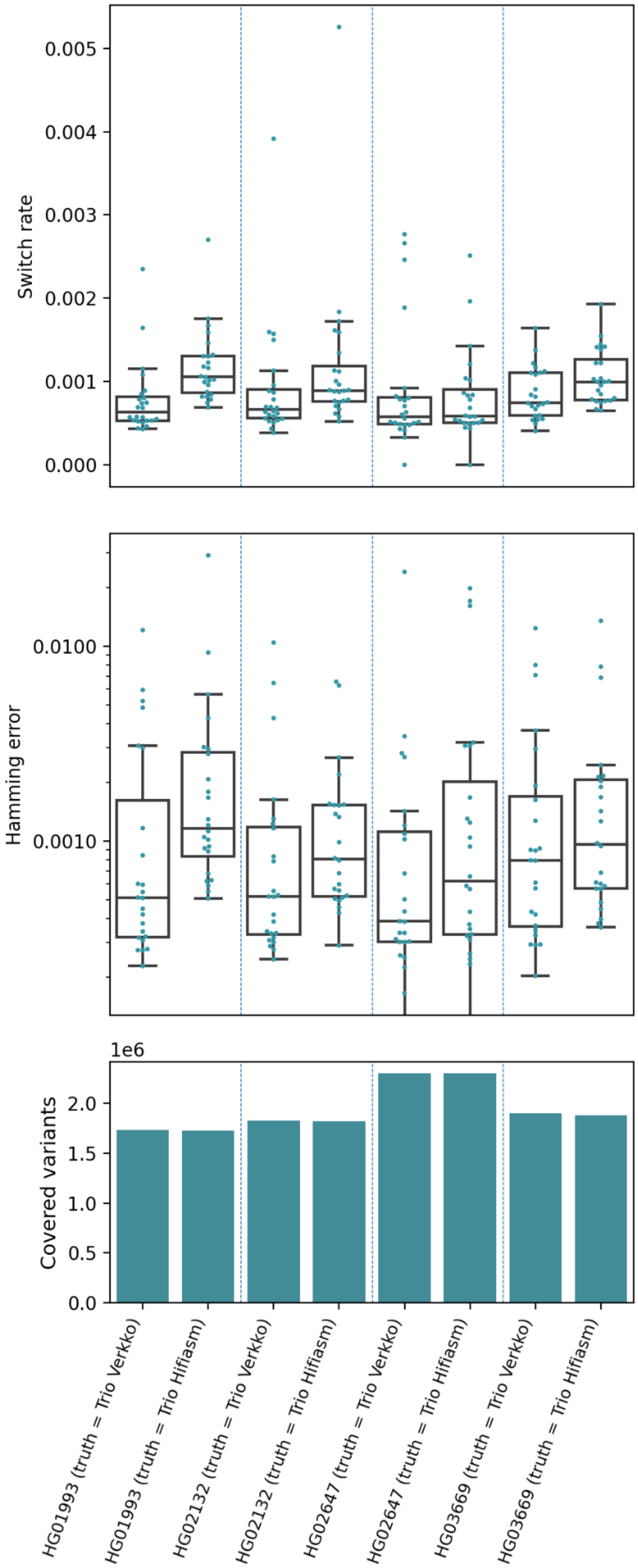

Supplementary Figure 3: HPRC evaluation using Trio Hifiasm and Trio Verkko aligned to HG38 as truth.

assembly	switch_rate	hamming_rate
hapdup_HG002_dual	0.0418	0.24119
shasta_hic_gfase_HG002_standard	0.11606	0.10936
shasta_trio_gfase_HG002_standard	0.1161	0.10727
shasta_hic_gfase_HG002_UL	0.09252	0.07675
shasta_trio_gfase_HG002_UL	0.09146	0.07736
shasta_hic_gfase_HG002_UUL	0.08471	0.07109
shasta_trio_gfase_HG002_UUL	0.08604	0.07304
shasta_hic_gfase_HG002_r10_1fc	0.05196	0.0537
shasta_trio_gfase_HG002_r10_1fc	0.05061	0.04522
shasta_hic_gfase_HG002_r10_ul_4fc	0.03234	0.0241
shasta_trio_gfase_HG002_r10_ul_4fc	0.03353	0.02557
shasta_hic_gfase_HG002_r10_uul_11fc	0.0336	0.0269
shasta_trio_gfase_HG002_r10_uul_11fc	0.03594	0.0285
verkko_gfase_HG002_full_coverage_v1_1	0.00639	0.00553
verkko_gfase_HG002_production_v1_1	0.00621	0.00521
verkko_trio_HG002_full_coverage	0.00846	0.00645
verkko_trio_HG002_production	0.00909	0.00639
hifiasm_gfase_HG002_r366	0.00643	0.0064
hifiasm_hic_HG002_v016	0.01105	0.01421
hifiasm_trio_HG002_v016	0.00822	0.00748

Supplementary Table 2: Evaluation of assemblies using yak Trio eval, comparing existing hybrid and trio assemblers to the HiC phased assemblies presented in this paper.

Variant Type	Shasta Confident Bed Variants	Verkko Confident Bed Variants	Shared Shasta Loci	Shared Verkko Loci	Shasta Only	Verkko Only	Shasta Indel	Shasta Indel Rate	Verkko Indel	Verkko Indel Rate	WhatsHap Covered Variants	WhatsHap Switch Rate	Whatshap Hamming Rate
0|1	1282348	1397762	1257356	1262499	24985	135255							
1|0	1283631	1399130	1258035	1263396	25593	135729							
1|2	42310	67573	38106	32437	4204	35136							
2|1	41251	67426	37137	31605	4114	35820							
Total Hets	2649540	2931891	2590634	2589937	58896	341940	34364	0.01297	256474	0.0869	2,483,238	0.000431	0.000347
Total Homs	2046423	1749922	1665743	1666205	380680	83717	342224	**0.16723**	30621	**0.0172**			

Supplementary Table 3: Investigation of Dipcall variants for the shasta_trio_gfase_HG002_r10_ul_4fc and verkko_trio_HG002_production assemblies. The assemblies share similar numbers of heterozygous and homozygous variant loci. However, compared to Shasta Dipcall variants the Verkko assembly has more variants, five times more assembly specific heterozygous variants, and seven times as many assembly specific heterozygous Indel variants. While the Shasta assembly has 4.5 times as many assembly specific homozygous variants, and 11 times as many assembly specific homozygous Indel variants compared to Verkko.
We ran WhatsHap Compare with the Verkko assembly as the truth set and Shasta as the query, the last three columns in the table. WhatsHap reports a blockwise hamming rate of 0.00035 for the 2.48 million Verkko heterozygous variants that agree in genotype with heterozygous Shasta Dipcall variants at the same loci.

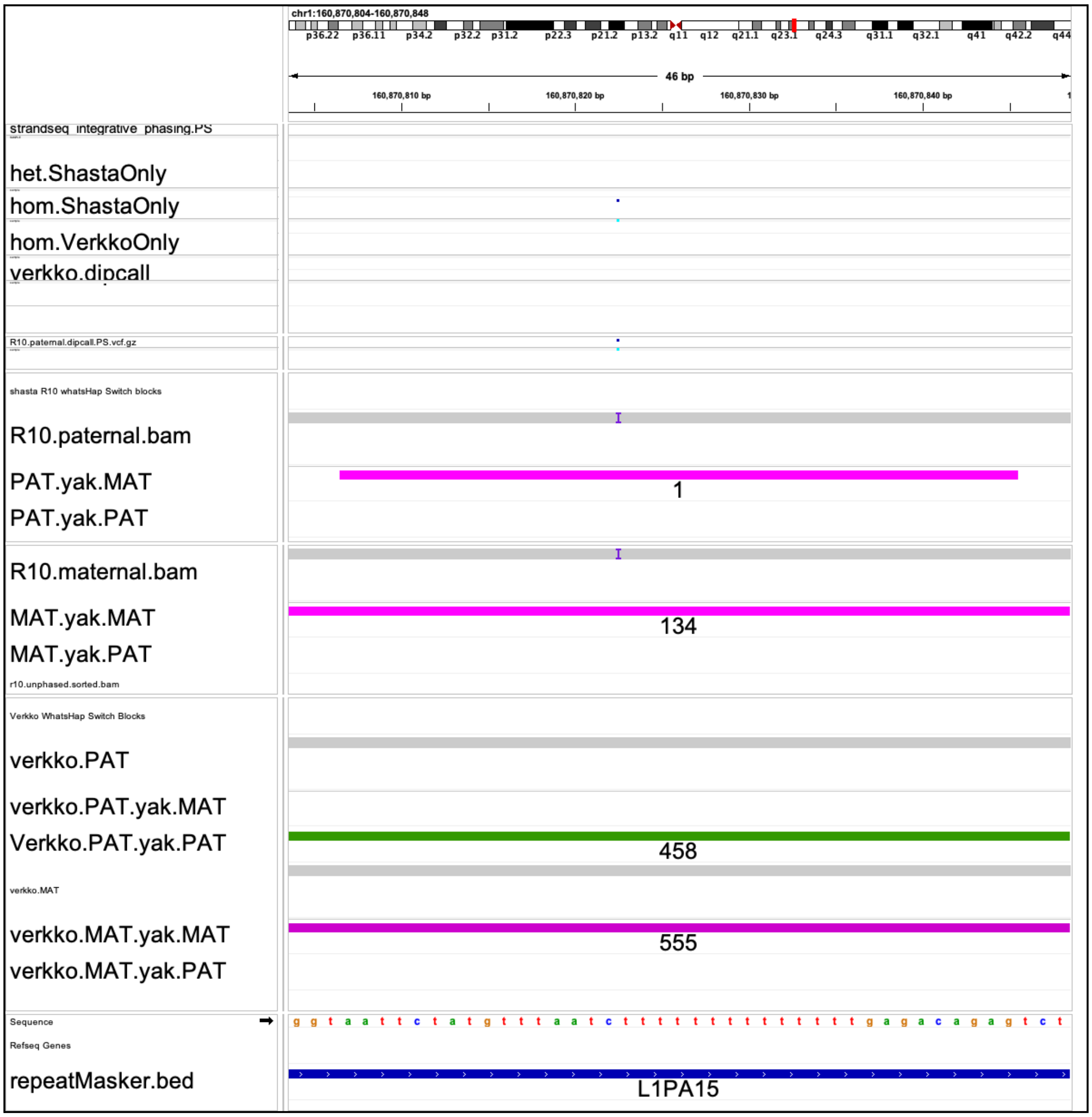

a)
b)

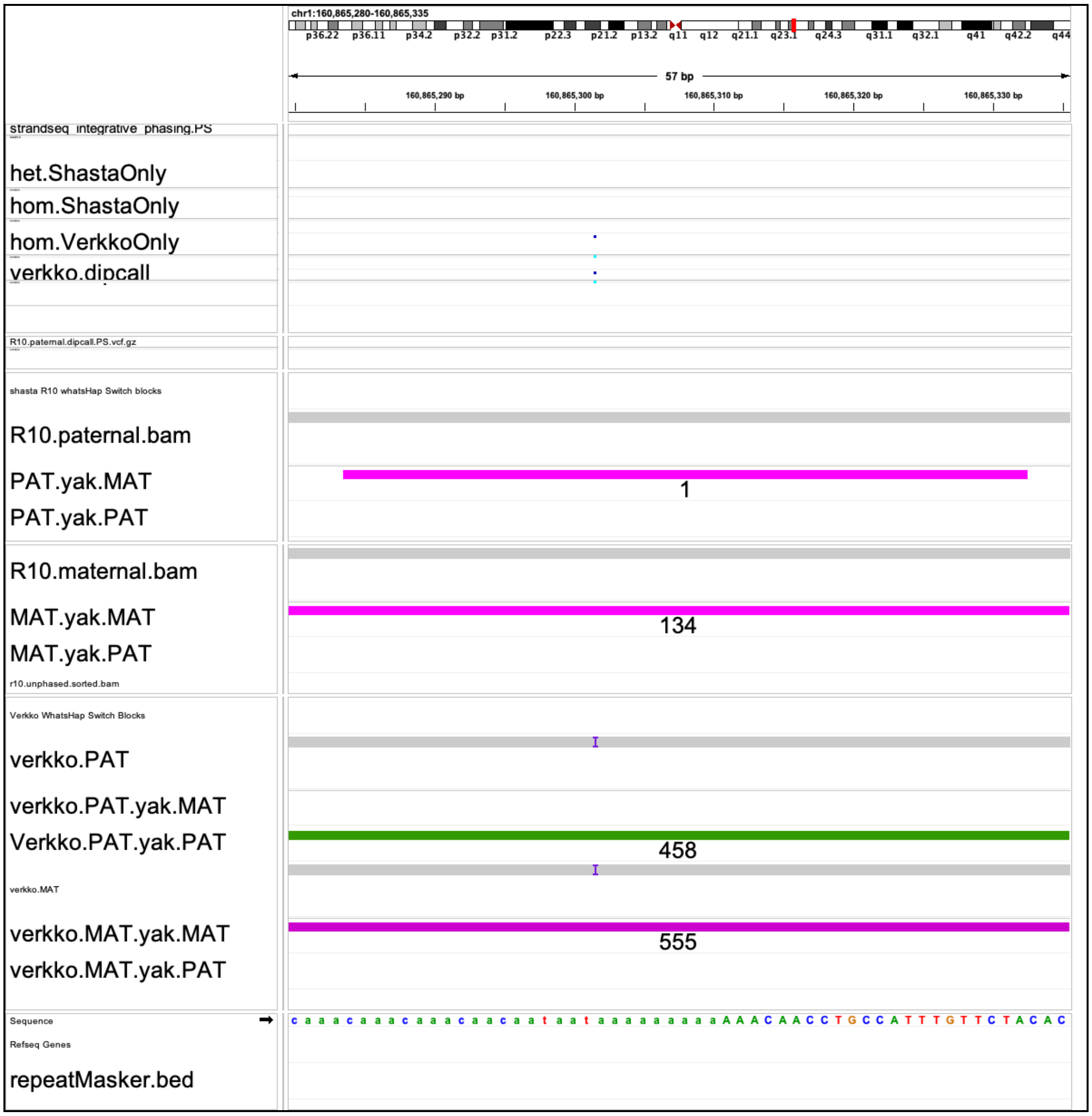
chr1:160,865,280-160,865,335
57 bp
160,865,290 bp
160,865,300 bp
160,865,310 bp
160,865,320 bp
160,865,330 bp
strandseq_integrative_phasing.PS
het.ShastaOnly
hom.ShastaOnly
hom.VerkkoOnly
verkko.dipcall
R10.paternal.dipcall.PS.vcf.gz
shasta R10 whatsHap Switch blocks
R10.paternal.bam
PAT.yak.MAT
1
PAT.yak.PAT
R10.maternal.bam
MAT.yak.MAT
134
MAT.yak.PAT
r10.unphased.sorted.bam
Verkko WhatsHap Switch Blocks
verkko.PAT
verkko.PAT.yak.MAT
Verkko.PAT.yak.PAT
458
verkko.MAT
verkko.MAT.yak.MAT
555
verkko.MAT.yak.PAT
Sequence
c a a a c a a a c a a a c a a c a a t a a t a a a a a a a a a A A A C A A C C T G C C A T T T G T T C T A C A C
Refseq Genes
repeatMasker.bed
c)

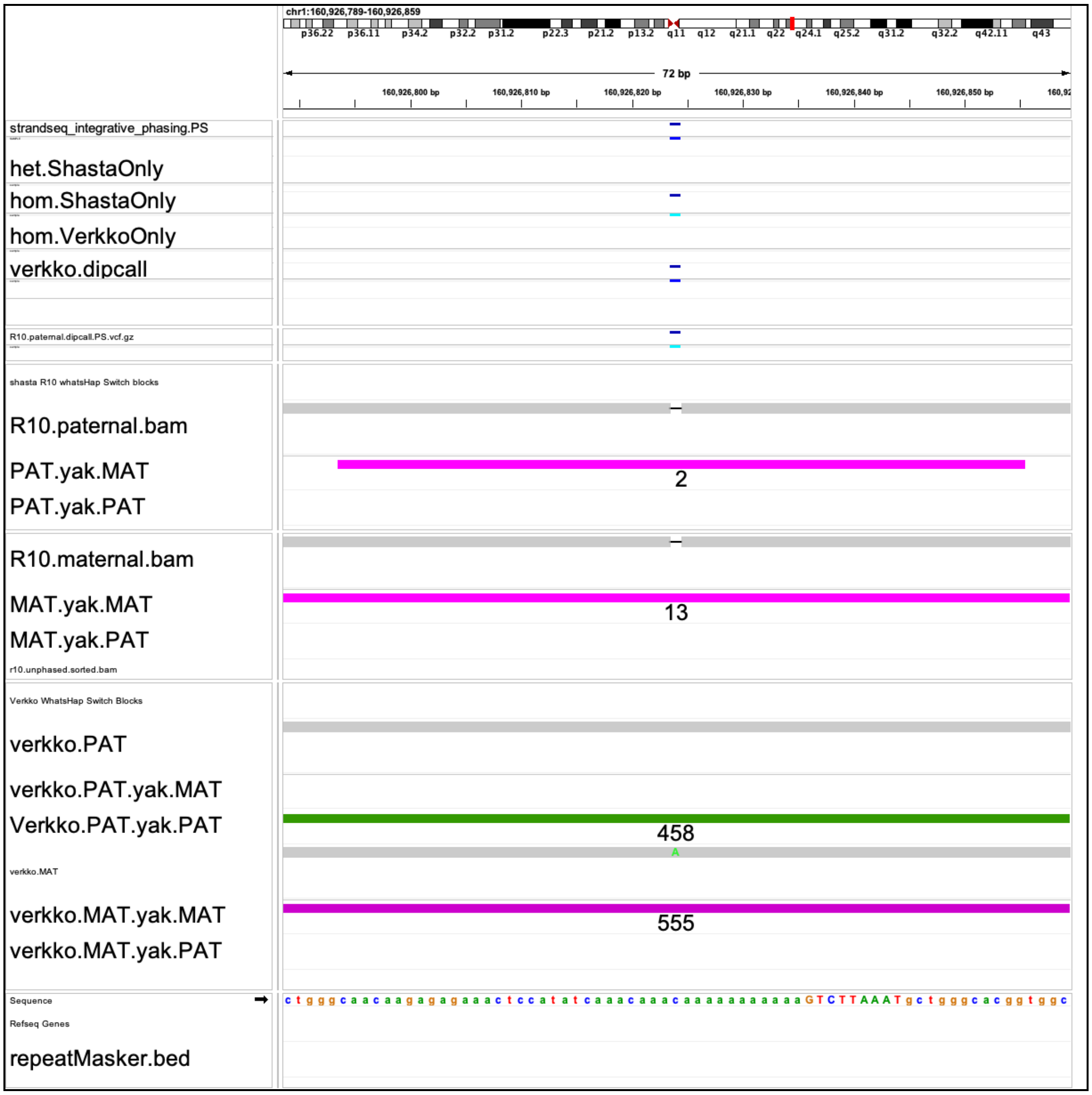

chr1:160,926,789-160,926,859
72 bp
160,926,800 bp
160,926,810 bp
160,926,820 bp
160,926,830 bp
160,926,840 bp
160,926,850 bp
strandseq_integrative_phasing.PS
het.ShastaOnly
hom.ShastaOnly
hom.VerkkoOnly
verkko.dipcall
R10.paternal.dipcall.PS.vcf.gz
shasta R10 whatsHap Switch blocks
R10.paternal.bam
PAT.yak.MAT
2
PAT.yak.PAT
R10.maternal.bam
MAT.yak.MAT
13
MAT.yak.PAT
r10.unphased.sorted.bam
Verkko WhatsHap Switch Blocks
verkko.PAT
verkko.PAT.yak.MAT
Verkko.PAT.yak.PAT
458
verkko.MAT
verkko.MAT.yak.MAT
555
verkko.MAT.yak.PAT
Sequence
ctgggcaacaagagagaaactccatatcaaacaaacaaaaaaaaaaaGTCTTAAATgctgggcacggtggc
Refseq Genes
repeatMasker.bed

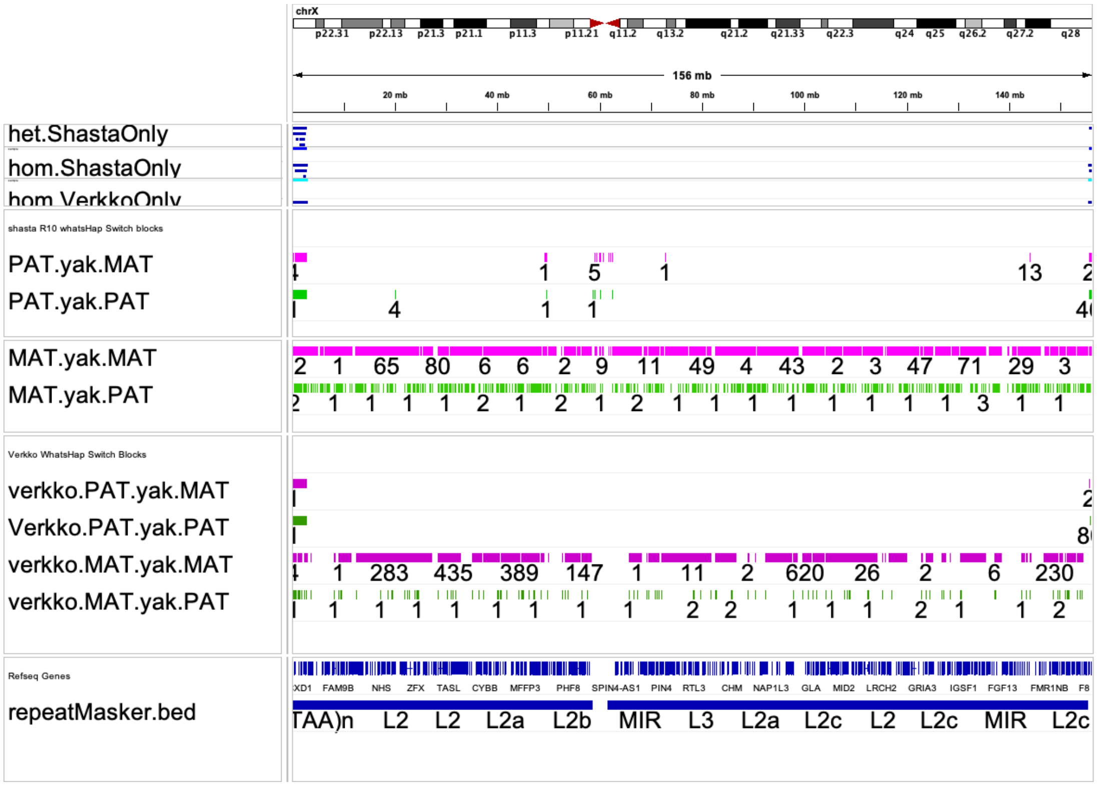

d)

Supplementary Figure 4: IGV screenshots of k-mer block locations where homopolymer Indel variants are matching with parental illumina k-mers, as reported by yak on and off target k-mer blocks. Shasta Assembly is shasta_trio_gfase_HG002_r10_ul_4fc, Verkko Assembly is verkko_trio_HG002_production. Magenta bed tracks are maternal yak k-mer blocks labeled with k-mer counts per block. Green bed tracks are paternal k-mer blocks with their respective k-mer counts labeled. a. Shasta specific homozygous “T” insertion in a homopolymer region creates a single maternal k-mer yak switch block in the paternal assembly. b. Verkko homozygous “A” insertion in a homopolymer region creates a single maternal k-mer yak switch block in the paternal assembly. c. A single maternal k-mer yak switch block in the paternal assembly is located at a truth variant. However, the genotypes between assemblies don’t match (homozygous “CA” deletion in Shasta, C-A SNP in Verkko) and as such wouldn’t be assessed by WhatsHap. d. Chromosome X illustrates a thicker lawn of off target green paternal k-mer matches in the Shasta yak blocks compared to the Verkko paternal yak k-mer blocks.
